# Genome-wide identification and characterization of long non-coding RNAs related to grain yield in foxtail millet [*Setaria italica* (L.) P. Beauv.]

**DOI:** 10.1186/s12864-020-07272-9

**Published:** 2020-12-01

**Authors:** Zilong Zhao, Dan Liu, Yanjiao Cui, Suying Li, Dan Liang, Daizhen Sun, Jianhe Wang, Zhengli Liu

**Affiliations:** 1 College of Agronomy, Shanxi Agricultural University, Taigu, China; 2 Department of Life Sciences, Tangshan Normal University, Tangshan, China; 3 Tianjin Key Laboratory of Crop Genetics and Breeding, Tianjin Crop Research Institute, Tianjin Academy of Agricultural Sciences, Tianjin, China

**Keywords:** Foxtail millet, Long non-coding RNA, Yield regulation

## Abstract

**Background:**

Long noncoding RNAs (lncRNAs) have been reported to play critical roles in diverse growth and development processes in plants. However, the systematic identification and characterization of lncRNAs in foxtail millet is nearly blank.

**Results:**

In this study, we performed high-throughput sequencing of young spikelets from four foxtail millet varieties in different yield levels at booting stage. As a result, a total of 12,378 novel lncRNAs were identified, and 70 were commonly significantly differentially expressed in comparisons between high-yield varieties and conventional varieties, suggesting that they involved in yield formation and regulation in foxtail millet. Functional analysis revealed that among the 70 significantly differentially expressed lncRNAs, 67 could transcriptionally modulate target genes in cis and in trans. Moreover, 18 lncRNAs related to grain yield in foxtail millet were predicted to function as miRNA target mimics and regulate gene expression by competing for the interaction between miRNAs and their target mRNAs.

**Conclusion:**

Our results will provide materials for elucidation of the molecular mechanisms of lncRNAs participate in yield regulation, and will contribute to high yield foxtail millet breeding.

**Supplementary Information:**

The online version contains supplementary material available at 10.1186/s12864-020-07272-9.

## Background

Long noncoding RNAs (lncRNAs) are generally defined as RNA transcripts that contain more than 200 nucleotides (nts) but lack a coding sequence (CDS) or open reading frame (ORF) [1]. Based on their genomic locations in relation to the neighboring protein-coding genes, lncRNAs can be divided into three classes: (1) long intergenic non-coding RNAs (lincRNAs), which are located within the genomic interval between two protein-coding genes; (2) intronic lncRNAs, which are derived from an intron; (3) long non-coding natural antisense transcripts (lncNATs), which are produced from the opposite strand and overlap with the exons [2].

According to their effects exerted on DNA sequences, lncRNAs can be classified to cis-acting lncRNAs and trans-acting lncRNAs, which regulate the expression of neighbouring genes and distant genes, respectively [3]. For example, the lncRNA Morrbid are reported to control the lifespan of eosinophils, neutrophils and monocytes in mouse, through recruiting the Polycomb repressive complex 2 (PRC2) at the promoter of nearby pro-apoptotic gene, *Bcl2l11*, and repressing its expression [4]. HOX ANTISENSE INTERGENIC RNA (HOTAIR), a 2.2 kb-long lncRNA derived from the human *HOXC* gene locus on chromosome 12, represses the transcription in trans of the *HOXD* gene locus on chromosome 2 spanning over 40 kb [5]. Recently, target mimicry has emerged as a new mechanism for regulating the function of microRNA (miRNA), which mediates the post-transcriptional regulation of the abundance of their mRNA targets through cleavage. INDUCED BY PHOSPHATE STARVATION 1 (IPS1) is an endogenous lncRNA identified from *Arabidopsis thaliana*. Pairing with a three-nucleotide bulge, *IPS1* binds to the phosphate starvation-induced miRNA miR399, and blocks the miR399-mediated cleavage effect of its target *PHO2* gene, resulting in increased accumulation of *PHO2* mRNA [6]. This evidence indicates that lncRNA is an important component of functional endogenous microRNA target mimics (eTMs). Aside from transcriptional and post-transcriptional regulation, lncRNAs may also function through other mechanisms, such as translation regulation, protein localization, telomere replication and RNA interference.

Until now, the well-studied lncRNAs are mainly from humans and animals, and they have been shown to be involved in multiple biological processes, including protein localization [7], cellular structure integrity [8], heat shock response [9], cell cycle [10, 11] and apoptosis [12, 13], and play a significant role in regulation of cancer progression and development of many other human diseases [14–17]. Although research in this field is far behind that in humans and animals, increasing lncRNAs have been identified in various plants, such as Arabidopsis, rice (*Oryza sativa* L.), maize (*Zea mays* L.), wheat (*Triticum aestivum* L.), with the rapid development of high-throughput RNA sequencing technology (RNA-seq). They may have roles in numerous growth and developmental processes, including root organogenesis [7], photo morphogenesis [18], flowering time [19] and flower development [20, 21], sexual reproduction [22, 23], fruit development and ripening [24, 25], and stress response [26–28]. However, the biological function of the majority of lncRNAs in plants and their regulatory roles remain largely unknown.

Foxtail millet [*Setaria italica* (L.) P. Beauv.], which originated from in China, has been a traditional cereal food crop since ancient times. According to its drought resistance, it does not require a lot of water input for growth. Moreover, the grain of foxtail millet is enriched in various amino acids and mineral nutrients, thus, foxtail millet has been one of the most important cereal crops in northern China, especially in arid area [29]. Due to its small genome size (490 Mb), inbreeding nature and genetic close relatedness to bioenergy feedstocks switchgrass (*Panicum virgatum*), napiergrass (*Pennisetum purpureum*) and pearl millet (*Pennisetum glaucum*), foxtail millet will serve as an important experimental model system [30–32]. Along with the extension and application of hybrids, foxtail millet yield has been largely increased. However, as lack of research on high yield molecular mechanism, the hybrid breeding of foxtail millet is entering the climbing up stage, and this seriously restricts the further improvement of foxtail millet yield. Therefore, elucidation of the mechanism of yield formation and regulation, and increasing the yield becomes an urgent issue to be solved in foxtail millet production.

As mentioned above, lncRNAs participate in multiple biological processes in plants. However, the reports on effects of lncRNAs on crop yield formation and regulation are relatively few. Wang et al. found that overexpression of a lncRNA generated from the antisense strand of neighbouring gene *leucine-rich repeat receptor kinase* (*LRK)* cluster, *LRK antisense intergenic RNA* (*LAIR*), increases rice grain yield and upregulates the expression of several *LRK* genes [33]. Zheng et al. found that 95% of differentially expressed lncRNAs identified in early developing panicles were significantly down-regulated in cultivated rice compared with wild rice. It was also shown that overexpression of three focal lncRNAs led to increased starch content and grain weight by using transgenic experiments and population analyses [34]. These results strongly indicated that lncRNAs have a role in regulation of rice grain yield. However, foxtail millet is mainly cultivated in developing countries, such as China and India, as a minor crop, therefore, identification and functional analysis of foxtail millet lncRNAs is still in its infancy, and no lncRNAs related to foxtail millet yield have been discovered so far.

In the present study, we firstly identified 12,378 novel lncRNAs by using the RNA-seq data derived from young spikelets of foxtail millet at booting stage. The structural characteristics and differential expression patterns of these lncRNAs were analyzed. We also predicted the potential function of lncRNAs. Our results implied that a number of lncRNAs contributed to yield formation and regulation in foxtail millet, and will provide new insights for investigation on the molecular mechanisms of lncRNAs. It was also indicated that lncRNAs may be useful targets for high yield foxtail millet breeding.

## Methods

### Plant materials

In this study, we used two foxtail millet conventional varieties, Jigu31 and Jigu32 (JG31 and JG32 for short, respectively), and two high-yield varieties, 5695 and 56229, whose yields were 17.03–26.30% higher than that of JG31 and JG32. The materials were planted at the Luannan Experiment Station of Foxtail Millet, Tangshan Normal University, Tangshan, China and Baixiang Experiment Station of Hebei Fengyuan Seed Industry Company, Xingtai, China in 2018 and 2019 for yield trait evaluation. Young spikelets at booting stage were sampled (Additional file 1: Fig. S1), and then frozen in liquid nitrogen. Three biological replicates were performed for each sample. The yield performance of four foxtail millet varieties was listed in Additional file 2: Table S1.

### Whole transcriptome library construction and high-throughput sequencing

The total RNA isolation, whole transcriptome libraries preparation and deep sequencing were performed by Beijing Ori-Gene Science and Technology (China). Transcriptome libraries were constructed using Ribo-Zero™ rRNA Removal Kits and NEBNext® Ultra Directional RNA Library Prep Kit for Illumina according to the manufacturer’s instructions. The libraries were sequenced initially on an HiSeq X Ten platform that generated paired-end reads of 150 bp.

### Transcriptome assembly

After removing the adaptors and low-quality reads from the raw data, we obtained the clean data. Efficient comparison between the clean reads and *Setaria italica* reference genome (*Sitalica*_312_v2.2) was performed by using HISAT2 [35], and the mapped reads were assembled using StringTie [36]. The RNA-seq saturation was measured using RSeQC [37].

### LncRNA identification

To obtain putative lncRNAs, we used a pipeline to filter the assembled novel transcripts through the following steps: (1) transcripts with single exons and transcripts shorter than 200 nt were removed; (2) four judgment methods, CPC (Coding Potential Calculator) [38], CNCI (Coding Non Coding Index) [39], PLEK (predictor of long non-coding RNAs and messenger RNAs based on an improved k-mer scheme) [40] and PFAM (protein families database) [41], were used to predict the coding potential of transcripts. If the positive transcripts were not common among the four tools, they would be removed. (3) according to their location, lncRNAs were classified to long intergenic lncRNAs (lincRNAs), intronic lncRNAs, antisense lncRNAs (lncNATs) and other lncRNAs.

### Differential expression analysis of lncRNAs

Gene expression FPKM values of lncRNAs were calculated with stringtie. The software edgeR [42] was used to test the differential expression of lncRNAs between the high-yield varieties and conventional varieties. The differentially expressed lncRNAs were chosen with a log_2_(fold change) ≥ 2 and false discovery rate (FDR) ≤ 0.01. Based on the normalized expression of lncRNAs from all samples, hierarchical clustering was performed using Cluster 3.0 to generate an overview of the characteristics of the expression profiles. Heatmaps were completed in R language.

### Prediction and functional analysis of target genes of differentially expressed lncRNAs

To predict the cis-target genes of lncRNA, we searched protein-coding genes 10 Kb upstream and downstream of lncRNA by BEDTools intersect [43]. To predict the trans-target genes, we calculated the Pearson’s correlation coefficients (R) between lncRNAs and mRNAs using R tool, and the genes with |R| ≥ 0.95 were identified as trans-target genes.

### Gene ontology (GO) enrichment analysis

GO enrichment analysis of target genes correlated with differentially expressed lncRNAs was implemented by the OmicShare tools (http://www.omicshare.com/tools).

### Prediction and functional analysis of lncRNA as miRNA precursor

To identify the lncRNAs function as precursors of miRNAs, the precursor sequences of known miRNAs in miRBase (http://www.mirbase.org) were aligned with lncRNAs identified in this study using BLAST. A lncRNA harboring a miRNA precursor sequence with 100% query identity and E-value less than 0.01 was defined as precursor of that miRNA. By bioinformatic analysis, the rest of the lncRNA sequences, whose structures accorded with the formation and action mechanism of miRNAs, were defined as precursors of novel miRNAs. The secondary structures of lncRNAs were plotted using RNAfold web server (http://rna.tbi.univie.ac.at/cgi-bin/RNAWebSuite/RNAfold.cgi).

### Interaction analysis of lncRNAs and mRNAs with miRNAs

The significantly differentially expressed miRNAs were selected with a |log_2_(fold change)| ≥ 1 and *p* < 0.05. The significantly differentially expressed mRNAs were selected with a |log_2_(fold change)| ≥ 2 and FDR < 0.01. Based on the co-expression analysis (p < 0.05), the interaction networks of significantly differentially expressed miRNAs, mRNAs and lncRNAs selected previously, which were common in four foxtail millet samples, were predicted. The lncRNA-miRNA-mRNA interaction networks were visualized with Cytoscape software [44].

### Quantitative real-time PCR validation

Six lncRNAs were randomly selected for quantitative real-time PCR (qRT-PCR) to validate the RNA-seq results. Total RNA extraction and cDNA synthesis were done as described previously [45]. qRT-PCR was performed using SuperReal PreMix Plus (SYBR Green, TIANGEN, China) on a QuantStudio 6 Flex Real-Time PCR system (Applied Biosystems, USA) following the manufacturer’s instructions. The PCR thermal profile included 95 °C for 15 s, and then 40 cycles of 95 °C for 10 s and 60 °C for 31 s. The relative expression level of each lncRNA was then calculated based on the eq. 2^−ΔΔCT^ [46]. All data were generated from averages of three independent replicates. All primers are listed in Additional file 4: Table S2.

## Results

### Overview of RNA sequencing data

To identity lncRNAs involved in foxtail millet grain yield formation and regulation, we constructed 12 (A1, A2 and A3 represented JG31; B1, B2 and B3 represented JG32; C1, C2 and C3 represented 5695; D1, D2 and D3 represented 56229) cDNA libraries from foxtail millet young spikelet samples at booting stages, and each sample was measured in three biological replicates. The libraries were sequenced using an Illumina HiSeq X Ten platform and a total of 1386.03 M raw reads were generated of 12 samples. After discarding adaptor sequences and low-quality reads, we obtained 1259.17 M clean reads. The percentage of clean reads in each library ranged from 82.82 to 92.98% (Table 1). Then, the clean reads were aligned to the *Setaria italica* reference genome (Sitalica_312_v2.2) by using HISAT2 [35], and approximately 76.94–90.59% of the clean reads were mapped to the reference genome. The junction saturation module from RSeQC [37] was applied to evaluate current sequencing depth (Additional file 3: Fig. S2). These results indicated that the quality of RNA-seq was good and our results were reliable (Table 2). A total of 161,406 novel transcripts were obtained after assembling of the mapped sequences in each library.

**Table 1:** Summary of RNA-seq data for young spikes of four foxtail millet varieties at booting stage

Sample	Raw reads (M)	Clean reads (M)	Percent	Clean bases (G)
A-1	91.893	83.580	90.95%	12.182
A-2	103.784	95.050	91.58%	13.823
A-3	83.007	75.437	90.88%	10.982
B-1	203.648	188.203	92.42%	27.543
B-2	105.759	87.592	82.82%	12.696
B-3	121.082	108.567	89.66%	16.007
C-1	109.232	101.561	92.98%	14.872
C-2	100.449	91.169	90.76%	13.119
C-3	95.082	87.542	92.07%	12.852
D-1	123.187	113.084	91.80%	16.486
D-2	122.901	112.051	91.17%	16.214
D-3	126.001	115.330	91.53%	16.806

The suffixes −1, −2, −3 indicate three biological replicates for each sample

**Table 2:** Summary of reads mapped to the *Setaria italica* reference genome

Sample	Total reads (M)	Total mapped (M)	Multiple mapped (M)	Uniquely mapped (M)
A-1	83.580	73.109 (87.47%)	0.000 (0.00%)	73.109 (87.47%)
A-2	95.050	83.747 (88.11%)	0.000 (0.00%)	83.747 (88.11%)
A-3	75.437	64.877 (86.00%)	0.000 (0.00%)	64.877 (86.00%)
B-1	188.203	158.339 (84.13%)	0.000 (0.00%)	158.339 (84.13%)
B-2	87.592	77.920 (88.96%)	0.000 (0.00%)	77.920 (88.96%)
B-3	108.567	98.352 (90.59%)	0.000 (0.00%)	98.352 (90.59%)
C-1	101.561	87.993 (86.64%)	0.000 (0.00%)	87.993 (86.64%)
C-2	91.169	73.553 (80.68%)	0.000 (0.00%)	73.553 (80.68%)
C-3	87.542	79.141 (90.40%)	0.000 (0.00%)	79.141 (90.40%)
D-1	113.084	99.289 (87.80%)	0.000 (0.00%)	99.289 (87.80%)
D-2	112.051	86.216 (76.94%)	0.000 (0.00%)	86.216 (76.94%)
D-3	115.330	100.840 (87.44%)	0.000 (0.00%)	100.840 (87.44%)

The suffixes −1, −2, −3 indicate three biological replicates for each sample

### Identification of lncRNAs in foxtail millet

**Fig. 1:**
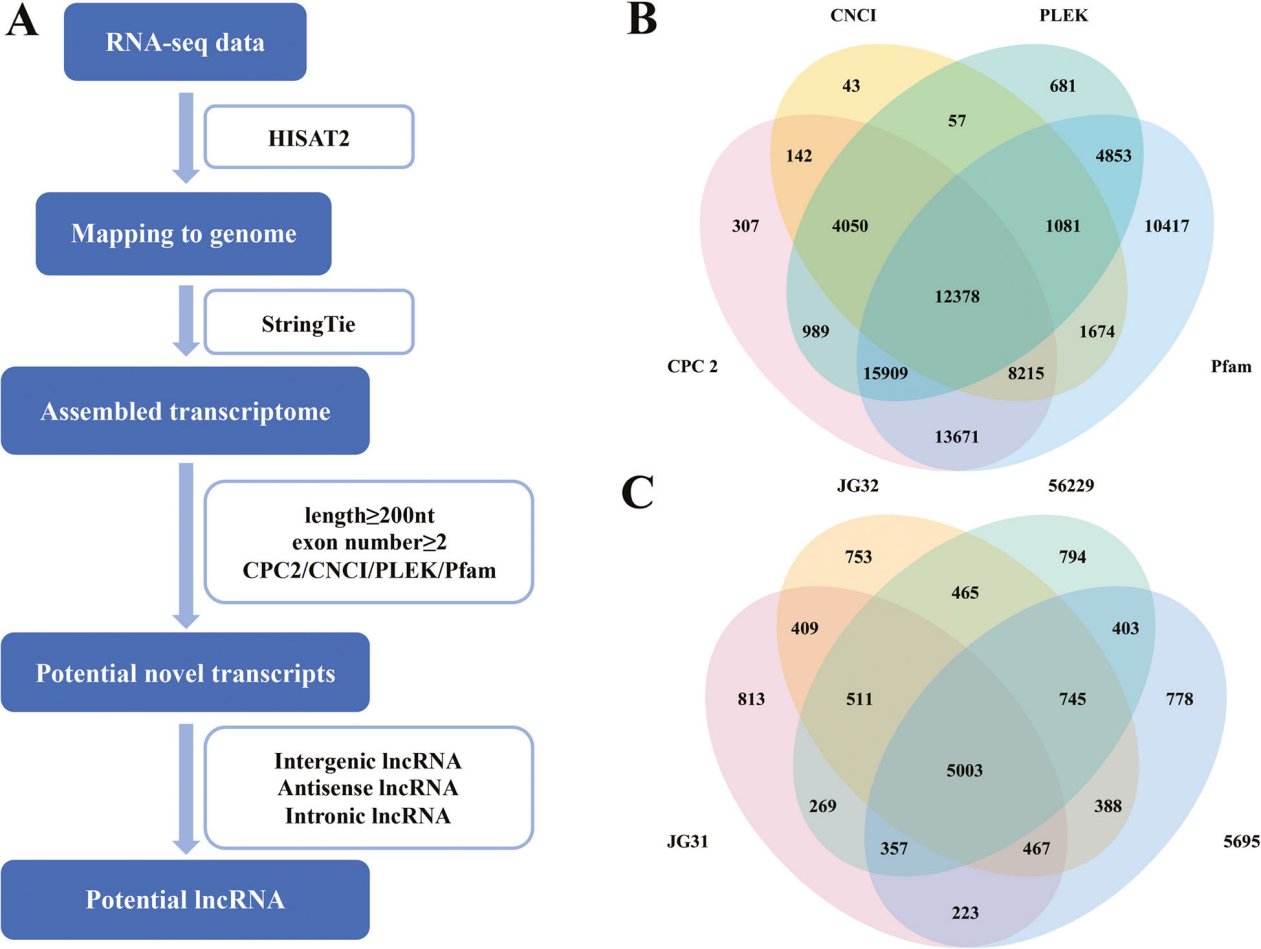
Identification of lncRNAs. **a** Identification pipeline for lncRNAs. **b** Protein-coding potential analysis using four methods. **c** The distribution of lncRNAs in four foxtail millet varieties

We developed a stringent filtering pipeline to discard transcripts without all the characteristics of lncRNA (Fig. 1a). After a basic filtering, the transcripts which had one exon and whose length were less than 200 nt were discarded. The software CPC2 [38], CNCI [39], PLEK [40] and Pfam database [41] were used to predict the protein-coding capacity of 161,406 novel transcripts simultaneously (Fig. 1b). As a result, a total of 12,378 lncRNAs were yielded from the young spikelets and used for further analysis, including 2091 lincRNAs (16.89%), 382 intronic lncRNAs (3.09%), 2314 lncNATs (18.69%) and 7591 other lncRNAs (61.33%), which could not be mapped to the accurate locations of the reference genome and classified into the above three types. In detail, 8052 lncRNAs in JG31, 8741 lncRNAs in JG32, 8364 lncRNAs in 5695 and 8547 lncRNAs in 56229 were obtained (Fig. 1c). In addition, 31,189 transcripts were identified as protein-coding transcripts.

**Fig. 2:**
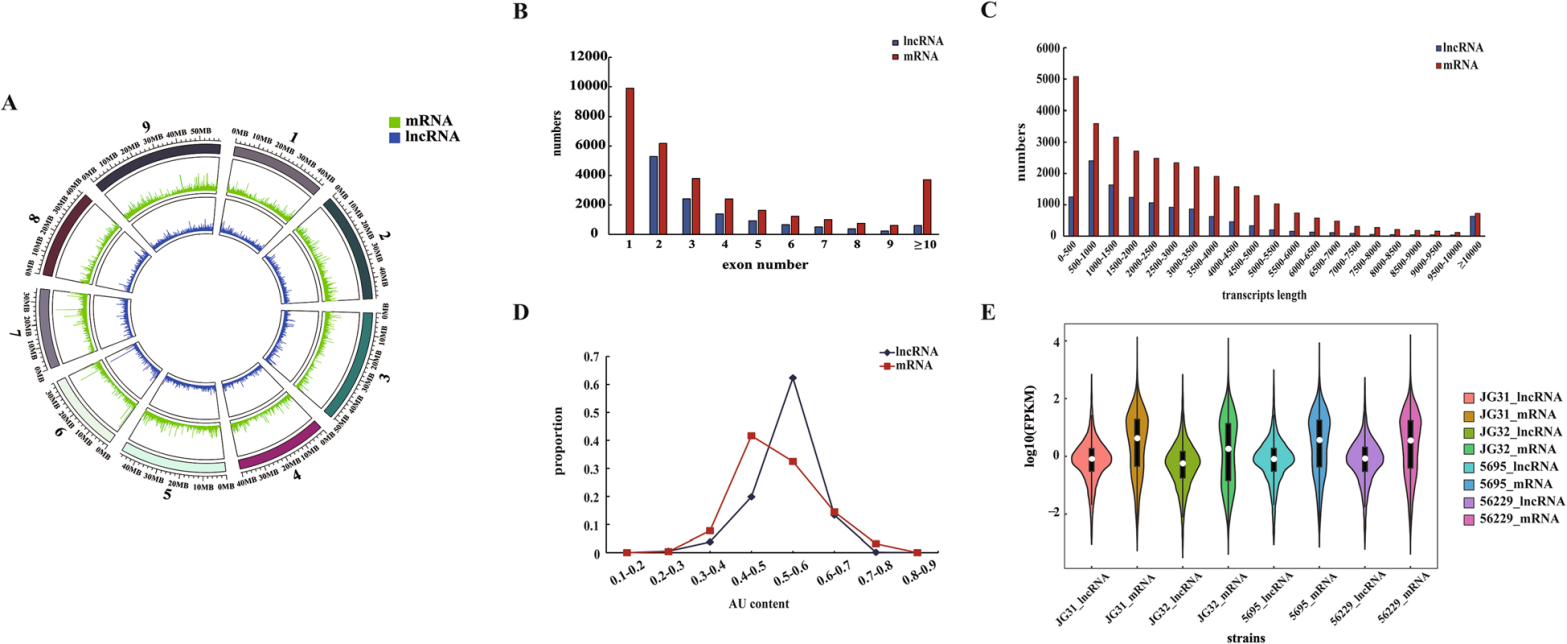
Properties of lncRNAs in foxtail millet young spikes. **a** Distribution of lncRNAs and protein-coding transcripts on nine chromosomes. **b** The number of exons per transcript for lncRNAs and protein-coding transcripts. **c** Transcript size for lncRNAs and protein-coding transcripts. **d** AU content of lncRNAs and protein-coding transcripts. **e** The violin plot of expression levels of lncRNAs and protein-coding transcripts. FPKM, fragments per kilobase of transcript per million fragments mapped

Circos plots [47] were used for visualization of the location of the obtained lncRNAs and protein-coding transcripts on foxtail millet chromosomes. We found that both lncRNAs and protein-coding transcripts were evenly distributed across nine chromosomes (Fig. 2a). The distribution tendencies of exon number and transcript length between lncRNAs and protein-coding transcripts were similar (Fig. 2b-c). The AU content of lncRNAs varied from 30 to 70%, with an average of 53.53%. However, in case of protein-coding transcripts, the AU content curve was flatter, with an average of 51.26% (Fig. 2d). We estimated the expression level of each transcript using fragments per kilobase of transcript per million fragments mapped (FPKM) and found that the expression levels of lncRNAs (median: 0.29 FPKM in JG31, 0.24 FPKM in JG32, 0.34 FPKM in 5695 and 0.36 FPKM in 56229) were relatively lower than the level at which protein-coding genes were expressed (median: 1.32 FPKM in JG31, 1.24 FPKM in JG32, 1.61 FPKM in 5695 and 1.52 FPKM in 56229) (Fig. 2e).

### Expression characterization of lncRNAs in foxtail millet varieties with different yield levels

**Fig. 3:**
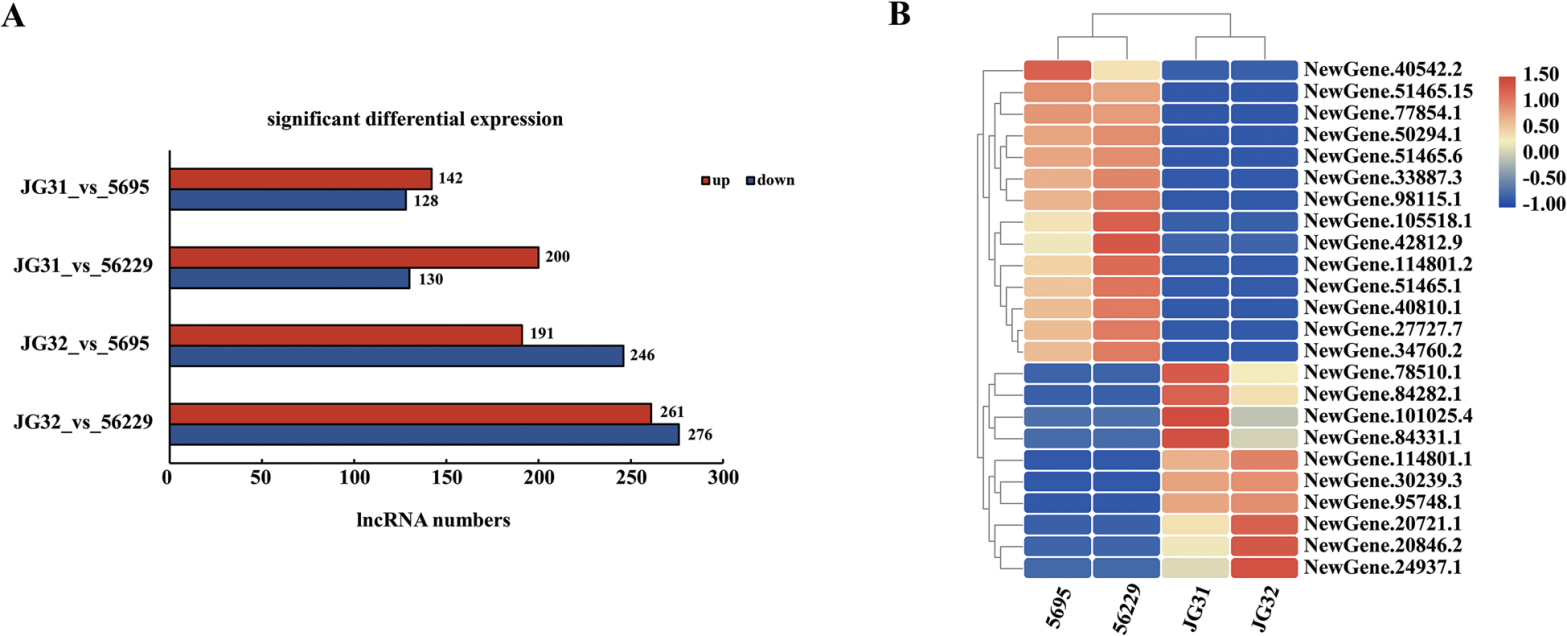
The profile of significantly differentially expressed lncRNAs in foxtail millet young spikes. **a** The distribution of significantly up-regulated and down-regulated differentially expressed lncRNAs in each comparison. **b** A heatmap was drawn to show the expression of 24 significantly differentially expressed lncRNAs commonly existed in each comparison. The color represented FPKM normalized log_2_transformed counts. Red indicated higher expression and blue indicated lower expression

To explore the lncRNAs involved in yield formation and regulation, we then detected the significantly differentially expressed lncRNAs between high-yield foxtail millet varieties (5695 and 56229) and conventional varieties (JG31 and JG32) using edgeR [42], and the lncRNAs showed a log_2_(fold change) ≥ 2 and false discovery rate (FDR) ≤0.01 were defined as significantly differentially expressed lncRNAs (Additional file 5: Table S3). As shown in Fig. 3a, 142 and 191 lncRNAs were found to be significantly up-regulated in variety 5695 when compared to JG31 and JG32, respectively. Meanwhile, 128 and 246 lncRNAs presented a significantly down-regulated pattern in 5695. Similarly, when compared to JG31 and JG32, 200 and 261 lncRNAs were up-regulated, 130 and 276 lncRNAs were down-regulated in 56229, respectively (Fig. 3a). We also found that 70 significantly differentially expressed lncRNAs were common in each comparison. Among the 70 lncRNAs, 24 lncRNAs were extracted randomly and their differentially expression profiles were demonstrated as a heatmap (Fig. 3b).

### GO enrichment analysis of target genes of yield-related lncRNAs

**Fig. 4:**
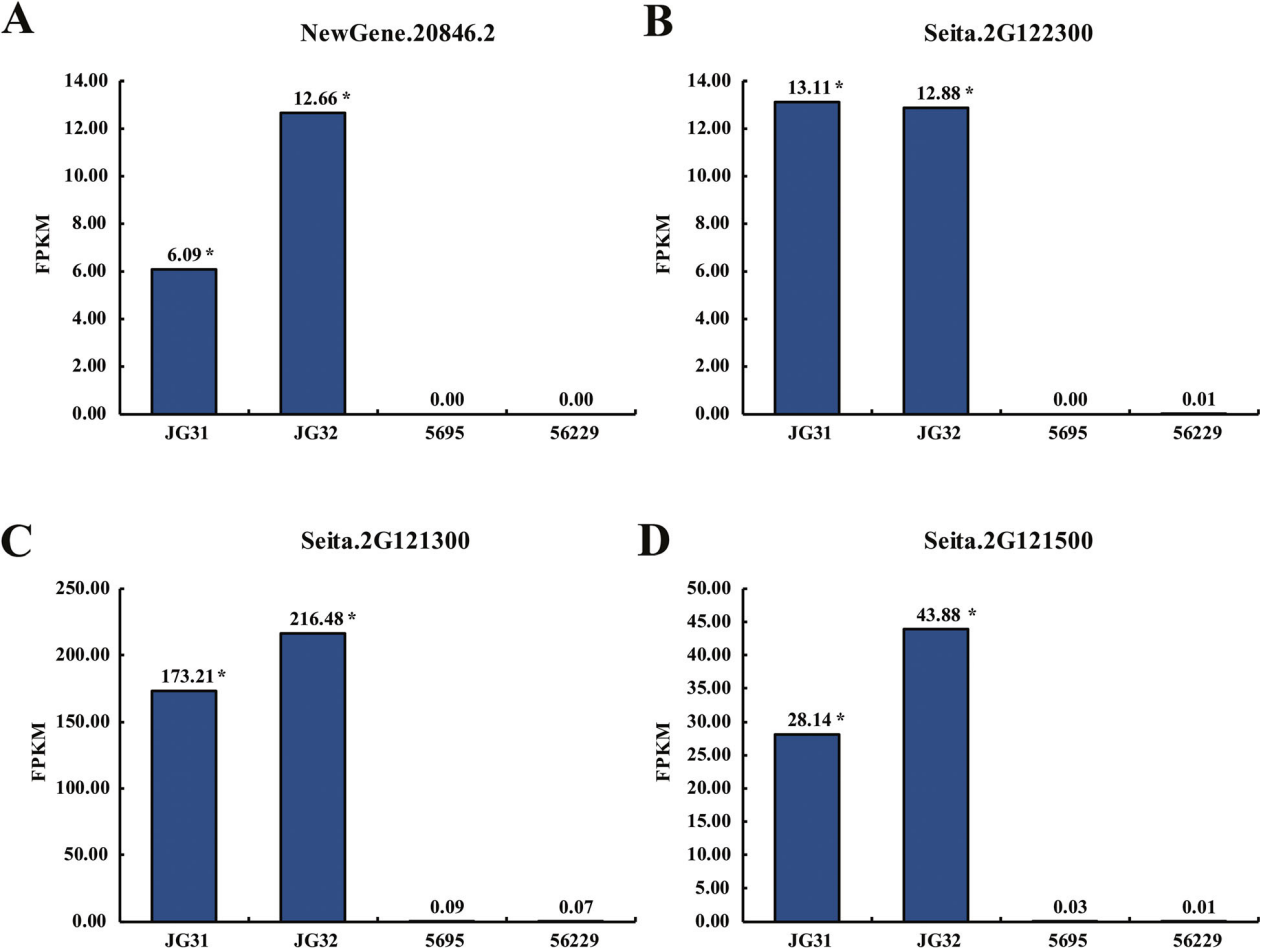
The expression levels of lncRNA NewGene.20846.2 and its three cis-regulated targets in RNA-seq data. The expression patterns of lncRNA NewGene.20846.2 (**a**) and its three cis-regulated targets, Seita.2G122300 (**b**), Seita.2G121300 (**c**) and Seita.2G121500 (**d**) in four foxtail millet varieties were shown. The asterisks indicated that the expression levels of these four transcripts in JG31 and JG32 were both significantly higher than that in 5695 and 56229 (*p* < 0.05). FPKM, fragments per kilobase of transcript per million fragments mapped

To further investigate the possible function of the 70 significantly differentially expressed lncRNAs in foxtail millet, we predicted their potential targets in cis-regulatory relationships. By searching for protein-coding genes located within 10 Kb upstream and downstream sites of these lncRNAs, 212 cis-target genes of 64 significantly differentially expressed lncRNAs were predicted. In addition, we identified targets of the 70 significantly differentially expressed lncRNAs in trans-regulatory network based on their expression correlation coefficient, and 3 trans-target genes of 3 significantly differentially expressed lncRNAs were identified. We found that most of the target genes (65.26%) showed concordant expression patterns with their significantly differentially expressed lncRNA partners in each comparison (Additional file 6: Table S4). For example, the expression level of lncRNA NewGene.20846.2 was significantly down-regulated in four comparisons (JG31 vs. 5695, JG31 vs. 56229, JG32 vs. 5695, JG32 vs. 56229 and the expression of its cis-regulated target genes, Seita.2G121300, Seita.2G122300 and Seita.2G121500 also changed in the same direction (Fig. 4).

**Fig. 5:**
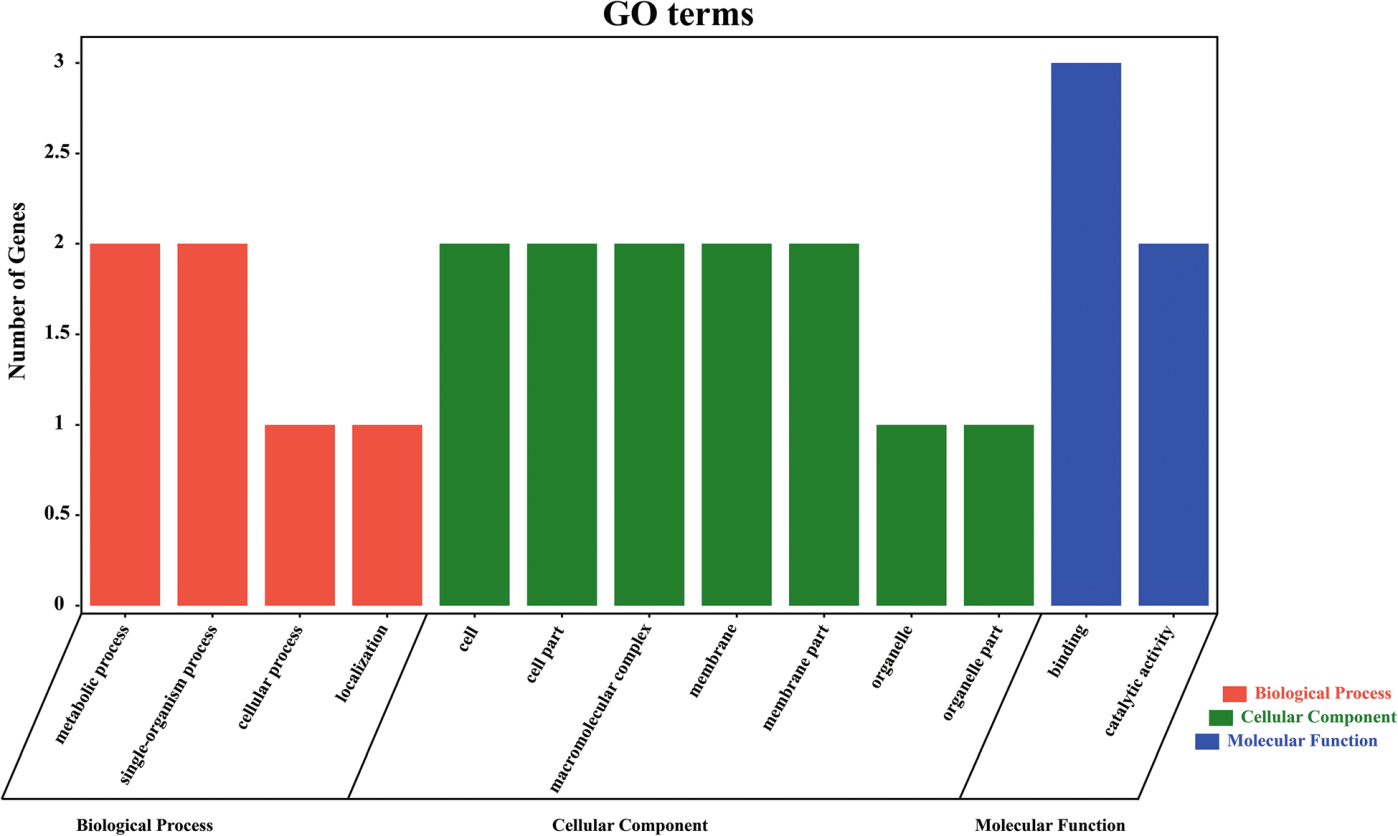
Gene ontology (GO) enrichment analysis of predicted target genes based on the significantly differentially expressed lncRNAs

Among the 215 target genes, 21 were significantly differentially expressed in all or partial comparisons (Additional file 6: Table S4). For example, the expression level of lncRNA NewGene.115177.1 was significantly down-regulated in four comparisons (JG31 vs. 5695, JG31 vs. 56229, JG32 vs. 5695, JG32 vs. 56229), and the expression of its predicted cis-regulated target genes Seita.8G131400 and Seita.8G131500, and trans-regulated target gene NewGene.115173, were also significantly decreased in four comparisons. The expression of lncRNA NewGene.24920.1 was significantly down-regulated in four comparisons, while the expression of its potential cis-regulated target gene Seita.2G182200 was only significantly increased in comparisons JG31 vs. 56229 and JG32 vs. 5695. Gene ontology (GO) analysis indicated that among the 21 target genes, 6 could be annotated to 13 GO terms, such as metabolic process (GO:0008152) and single-organism process (GO:0044699) in biological process section, cell (GO:0005623) and cell part (GO:0044464) in cellular component section, and catalytic activity (GO:0003824) and binding (GO:0005488) in molecular function section (Fig. 5, Additional file 6: Table S4). These results implied that the target genes of significantly differentially expressed lncRNAs in young spikelets of foxtail millet function in multiple biological processes.

### Identification of yield-related lncRNAs as potential miRNA precursors

**Fig. 6:**
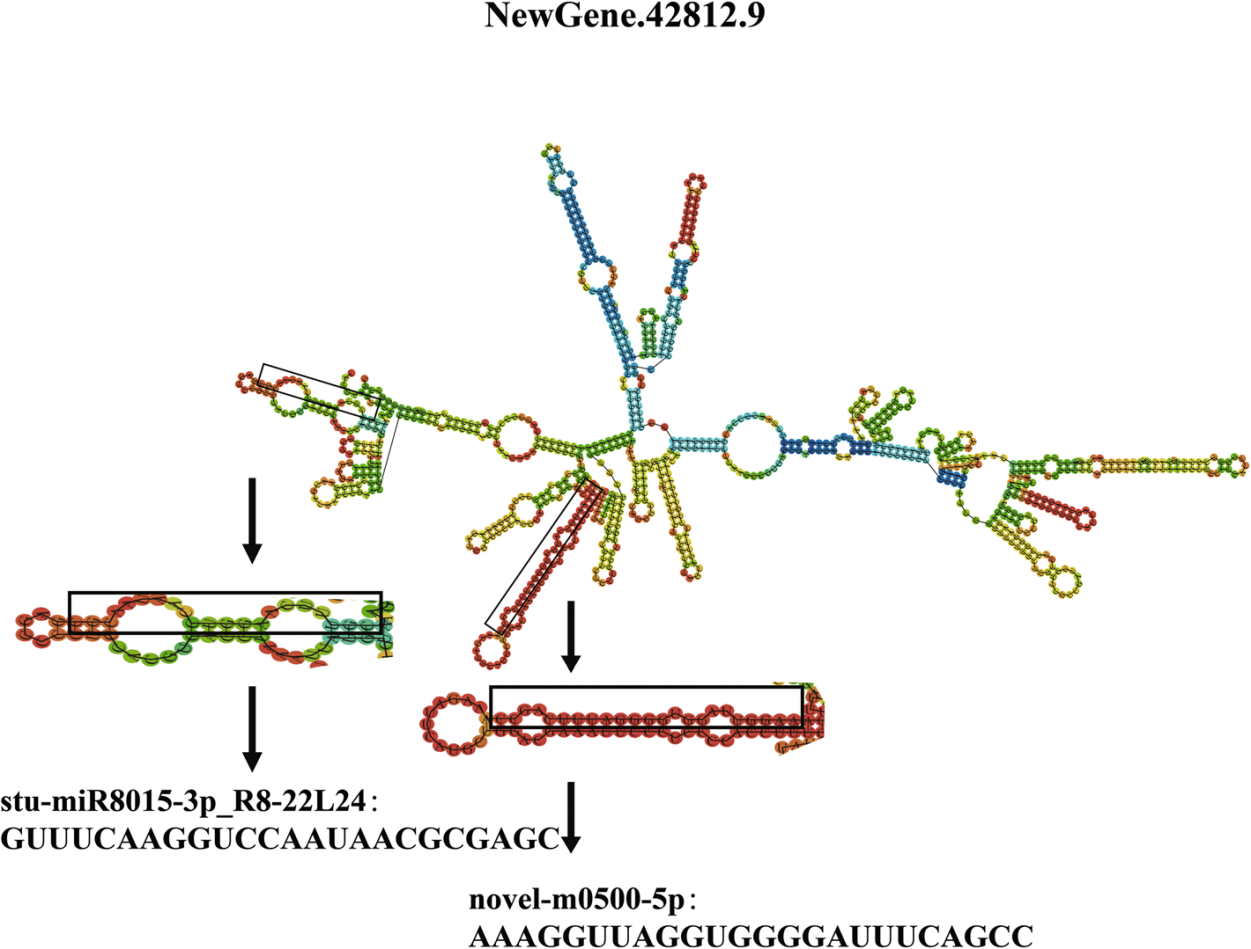
The secondary structure of lncRNA (NewGene.42812.9), which contained putative precursors of two miRNAs novel-m0500-5p and stu-miR8015-3p_R8-22L24

LncRNAs can also serve as precursors for small RNA biosynthesis, including small nuclear RNAs (snRNAs), small nucleolar RNAs (snoRNAs) and miRNAs [48]. Among the 70 significantly differentially expressed lncRNAs in foxtail millet, only one lncRNA NewGene.42812.9, which was significantly up-regulated in high-yield foxtail millet varieties 5695 and 56229 when compared to conventional varieties JG31 and JG32, was predicted as precursor of two different miRNAs. As shown in Fig. 6, the mature miRNAs, novel-m0500-5p and stu-miR8015-3p_R8-22L24, might be generated via cleavage of NewGene.42812.9 with stem-loop by an endonuclease.

### Identification of yield-related lncRNAs acting as target mimic of miRNAs

**Fig. 7:**
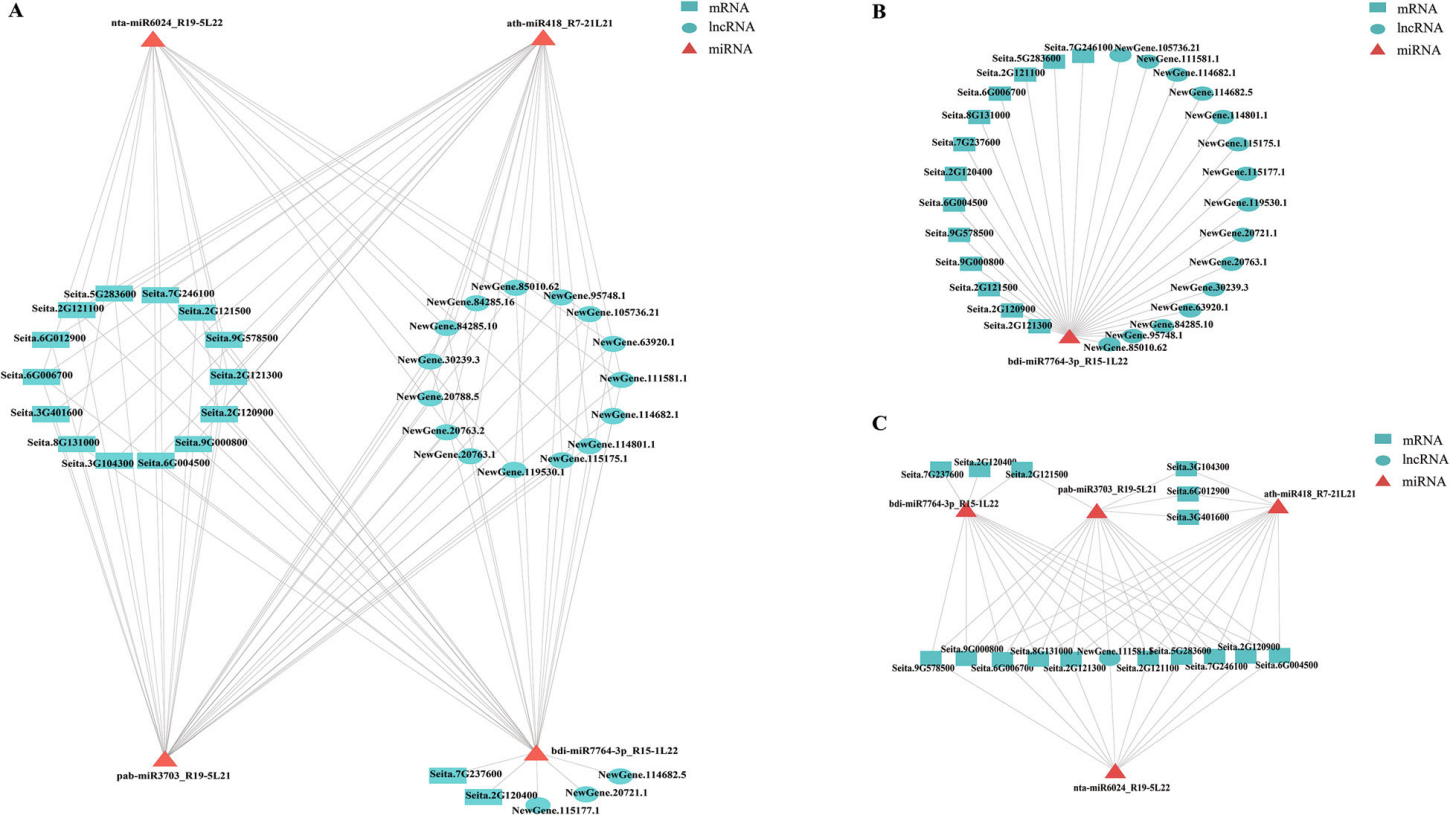
The interaction network of significantly differentially expressed lncRNAs, miRNAs and mRNAs. **a** The interaction network of four miRNAs with 16 mRNAs and 18 lncRNAs. **b** The interaction network of miRNA bdi-miR7764-3p_R15-1 L22 with 15 lncRNAs and 13 mRNAs. **c** The interaction network of four miRNAs with 16 mRNAs and one lncRNA NewGene.111581.1. The red triangles indicate upregulated miRNAs, the green ovals and rectangles represent down-regulated lncRNAs and miRNAs in spikes of high-yield foxtail millet varieties

To further examine the potential regulatory function of lncRNAs related to foxtail millet grain yield, an interaction analysis among significantly differentially expressed lncRNAs, miRNAs and mRNAs was performed (Fig. 7a, Additional file 7: Table S5). Among the 70 significantly differentially expressed lncRNAs in foxtail millet, a total of 18 lncRNAs showed interaction with four miRNAs, which were further interacted with 16 mRNA transcripts, suggesting that these 18 lncRNAs may act as miRNA target mimics, competing for binding to miRNAs and preventing the binding of miRNAs to target mRNAs. In the expression interaction network, single miRNA could bind to several lncRNAs and mRNAs. For example, miRNA bdi-miR7764-3p_R15-1 L22 had 28 target sites including 15 lncRNAs and 13 mRNAs (Fig. 7b). Moreover, multiple miRNAs could interact with single lncRNA or mRNA. For example, all the four significantly differentially expressed miRNAs could be targeted by lncRNA NewGene.111581.1 (Fig. 7c). In addition, another set of interaction was also detected, that is one lncRNA or mRNA could only pair with one single miRNA. For example, three lncRNAs, NewGene.115177.1, NewGene.20721.1 and NewGene.114682.5, and two mRNAs, Seita.7G237600 and Seita.2G120400, could only interact with miRNA bdi-miR7764-3p_R15-1 L22 (Fig. 7a). We also found that all the four significantly differentially expressed miRNAs in the interaction network were upregulated, and lncRNAs and mRNAs, which the four miRNAs interacted with, were downregulated in high-yield foxtail millet varieties, 5695 and 56229. The results implied that lncRNAs may regulate the activity of miRNAs by acting as target mimics and play important roles in foxtail millet yield formation and regulation.

### Validation of the significantly differentially expressed lncRNAs by qRT-PCR

**Fig. 8:**
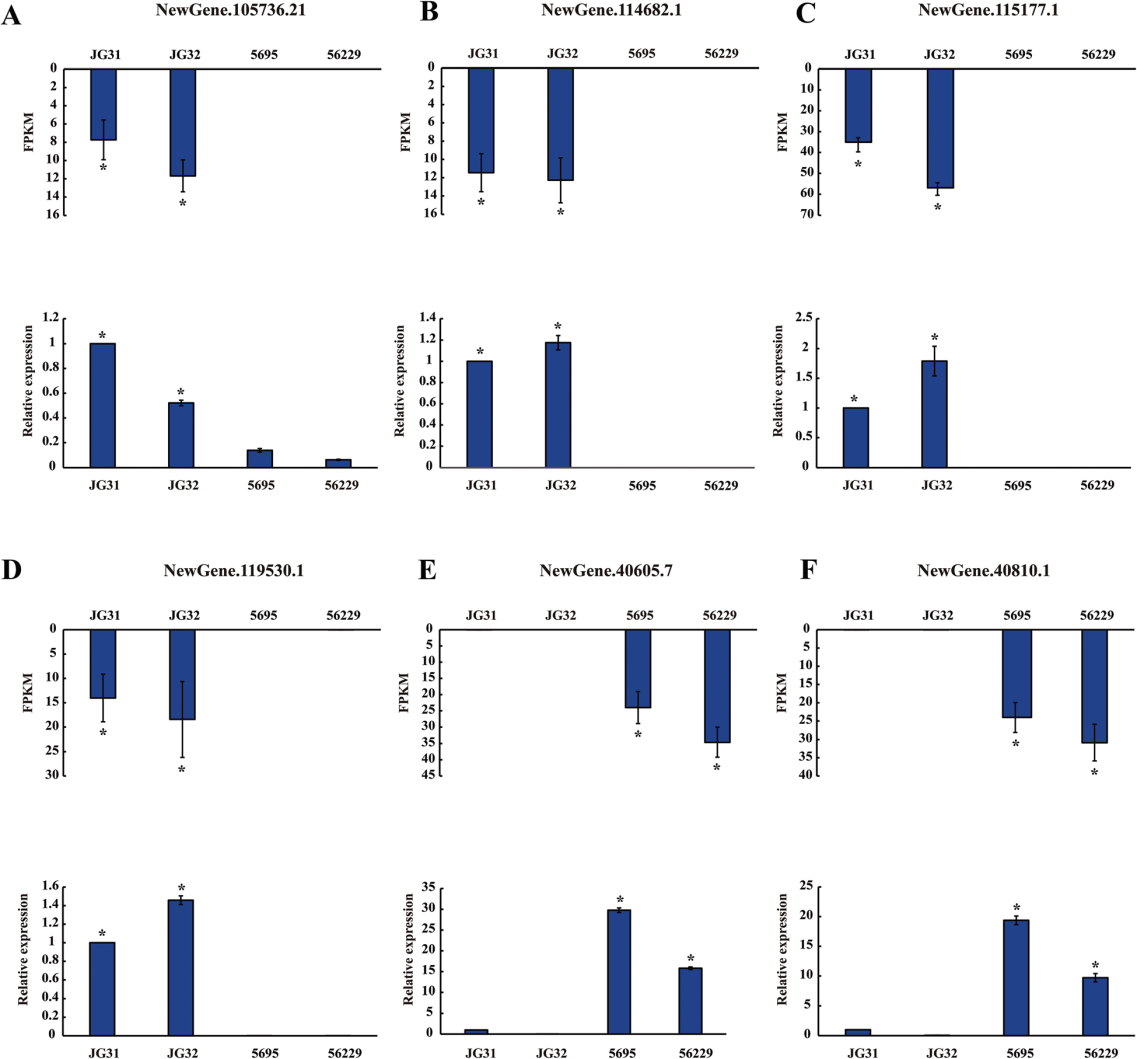
Validation of six significantly differentially expressed lncRNAs by qRT-PCR. The expression patterns of six lncRNAs NewGene.105736.21 (**a**), NewGene.114682.1 (**b**), NewGene.115177.1 (**c**), NewGene.119530.1 (**d**), NewGene.40605.7 (**e**) and NewGene.40810.1 (**f**) in RNA-seq data (top panel) and qRT-PCR results (bottom panel) were shown. The *SiACTIN* gene was used as an internal control. The asterisks indicated that the expression levels of these lncRNAs in JG31 and JG32 were both significantly higher or lower than that in 5695 and 56229 (*p* < 0.05). FPKM, fragments per kilobase of transcript per million fragments mapped. Error bars represent SD

To verify the RNA-seq results, six predicted significantly differentially expressed lncRNAs were randomly chosen to perform qRT-PCR. As shown in Fig. 8, the expression levels of NewGene.105736.21, NewGene.114682.1, NewGene.115177.1 and NewGene.119530.1 were significantly down-regulated in 5695 and 56229 compared to that in JG31 and JG32, whereas the expression levels of NewGene.40605.7 and NewGene.40810.1 were significantly up-regulated in 5695 and 56229 compared to that in JG31 and JG32. These results were consistent with the sequencing results, indicating the accuracy of our RNA-seq analysis and further supporting the role of these lncRNAs in foxtail millet yield formation and regulation.

## Discussion

In the past decade, amounts of researches revealed the critical roles of lncRNAs in growth and development processes of plants. Although thousands of lncRNAs have been characterized in model angiosperm plants, such as Arabidopsis, rice, maize, wheat and soybean, the research on foxtail millet lncRNAs is nearly blank. In this study, we performed a genome-wide identification of lncRNAs using RNA-seq data obtained from four foxtail millet varieties in different yield levels, and a total of 12,378 novel lncRNAs were identified in the young spikelets of foxtail millet at booting stage, providing materials for the study of yield formation and regulation in foxtail millet. Furthermore, the structures and expression patterns of these lncRNAs were analyzed, and it was shown that the structural characteristics and expression profiles were in agreement with previous studies [22, 28]. For example, the expression levels of lncRNAs were generally lower than that of protein-coding genes, and the lncRNAs were AU rich as compared to the coding sequences in foxtail millet (Fig. 2), indicating that these features of lncRNAs were conserved in various plant species.

The early developing spikelet tissue plays an important role in determining the grain size, grain number and grain quality [49–51], so we collected young spikelets at booting stage as samples to characterize the lncRNA transcriptome and explore its function in foxtail millet yield. After a differential expression analysis, 270, 330, 437 and 537 lncRNAs revealed significant differential expression in four comparisons between high-yield foxtail millet varieties (5695 and 56229) and conventional varieties (JG31 and JG32), respectively (Fig. 3), suggesting that lncRNAs may involve in yield formation and regulation in foxtail millet. Then, we found that these lncRNAs could transcriptionally regulate target genes in cis and in trans (Additional file 6: Table S4, Fig. 4), implied that the significantly differentially expressed lncRNAs in young spikelets at booting stage might regulate grain yield through modulating the expression levels of their target genes. However, as these targets regulated by lncRNAs were largely functionally unknown, further investigations are needed to fully elucidate the exact regulatory mechanisms.

Some lncRNAs can act as precursors for the biogenesis of miRNAs to silence target mRNAs [48]. For example, 26 significantly differentially expressed lncRNAs could act as the precursors of certain miRNAs and may involve in the ovule development and female gametophyte fertility in rice [23]. In bread wheat, 19 lncRNAs were predicted as precursor of 28 miRNAs during development and stress response [28]. In this study, lncRNA NewGene.42812.9 was predicted as precursor of miRNAs novel-m0500-5p and stu-miR8015-3p_R8-22L24 (Fig. 6). However, we found that the expression trend of novel-m0500-5p was not consistent with NewGene.42812.9, and stu-miR8015-3p_R8-22L24 was not significantly differentially expressed (Additional file 8: Fig. S3). This result indicated that lncRNAs may not work as miRNA precursors and regulate target mRNAs at a post-transcriptional level during yield formation and regulation in foxtail millet.

Several studies showed that lncRNAs can act as target mimics of miRNAs and regulate gene expression, by interfering with the interaction between miRNAs and their target mRNAs. In the current study, a total of 18 significantly differentially expressed lncRNAs were predicted to function as miRNA target mimics and regulate gene expression by binding to miRNAs in competition with mRNAs (Fig. 7, Additional file 7: Table S5). For example, nta-miR6024_R19-5 L22, a miRNA in foxtail millet, was found to interact with 7 lncRNAs and 9 mRNAs (Fig. 6), and the expression levels of lncRNAs were inversely proportional to that of nta-miR6024_R19-5 L22 (Additional file 9: Fig. S4). According to previous researches, miR6024 plays a crucial role in responses to biotic and abiotic stresses. The sly-miR6024 has been documented to be involved in drought response in tomato (*Solanum lycopersicum*), and its expression level is down-regulated in drought-sensitive cultivar, and up-regulated in drought-tolerant introgression line [52]. Ethylene is one of the most important plant hormones, which participate in many biological processes, including responses to biotic and abiotic stresses. High-throughput sequencing and bioinformatics analysis reveal that miR6024 plays a role in ethylene biosynthesis and signal transduction pathway [53]. In addition, miR6024 was also annotated as an microRNA related to disease resistance. *Potato spindle tuber viroid* (PSTVd) is a species of Pospiviroidae family and infects Solanaceae and Asteraceae (or Compositae) families. The level of miR6024 is high in stem tissues of tomato plants infected with PSTVd-I (Intermediate), a new PSTVd variant induces severe symptoms, and is low in stem tissues of tomato plants infected with PSTVd-D (Dahlia), a PSTVd variant induces very mild symptoms [54]. As we all know, environmental stresses, such as heat, drought and salt, can seriously affect crop yield. Therefore, we speculate that lncRNAs may act as miRNA target mimics and involve in yield formation and regulation by adjusting the responses to abiotic and biotic stresses in foxtail millet. However, further studies are needed to elucidate the mechanism.

## Conclusions

By using RNA-seq data derived from four foxtail millet varieties in different yield levels, we identified 12,378 novel lncRNAs in young spikelets of foxtail millet at booting stage. The characteristics and differential expression patterns of these lncRNAs were analyzed, and 70 significantly differentially expressed lncRNAs were considered to be closely related with grain yield in foxtail millet. Further functional analysis indicated that these lncRNAs may involve in yield regulation by transcriptionally modulating cis-target genes and trans-target genes, or acting as target mimics of miRNAs. These results will provide a source for investigation on important functions and regulatory mechanisms of lncRNAs in foxtail millet yield formation and regulation.

## Supplementary Information

**Additional file 1: Fig. S1.** The young spikelets of four foxtail millet varieties at booting stage were laid out.

**Additional file 2: Table S1.** Yield performance of four foxtail millet varieties used in this study.

**Additional file 3: Fig. S2.** The saturation analysis of lncRNAs from four foxtail millet varieties. The three biological replicates of RNA-seq saturation for lncRNAs from JG31 (A), JG32 (B), 5695 (C) and 56229 (D) were shown. X axis is the percentage of total reads resampled. Y axis is the number of detected splicing junctions.

**Additional file 4: Table S2.** Summary of significantly differentially expressed lncRNAs.

**Additional file 5: Table S3.** The target genes of significant differentially expressed lncRNAs in all comparisons (GO annotation of these genes were listed). * indicated the significantly differentially expressed target genes.

**Additional file 6: Table S4.** The network of significantly differentially expressed lncRNAs, miRNAs and mRNAs.

**Additional file 7: Table S5.** The primers used for quantitative real-time PCR.

**Additional file 8: Fig. S3.** The expression levels of two miRNAs and their predicted lncRNA precursor. The lncRNA NewGene.42812.9 (A) was predicted precursor of miRNAs novel-m0500-5p (B) and stu-miR8015-3p_R8-22L24 (C). FPKM, fragments per kilobase of transcript per million fragments mapped. RPM, reads per kilobase of transcript per million mapped.

**Additional file 9: Fig. S4.** The expression levels of miRNA and its predicted target mimics. The lncRNAs NewGene.84285.10 (B), NewGene.111581.1 (C), NewGene.114801.1 (D), NewGene.63920.1 (E), NewGene.20763.1 (F), NewGene.119530.1 (G) and NewGene. 105,736.21 (H) were predicted target mimics of miRNA nta-miR6024_R19-5 L22 (A). FPKM, fragments per kilobase of transcript per million fragments mapped. RPM, reads per kilobase of transcript per million mapped.

## Data Availability

All sequence data has been submitted to Sequence Read Archive (SRA) of the National Center for Biotechnology Information (NCBI) under the accession number PRJNA655982 (https://www.ncbi.nlm.nih.gov/sra/PRJNA655982).

## Abbreviations

lncRNA: Long noncoding RNA
CDS: Coding sequence
ORF: Open reading frame
lincRNA: Long intergenic non-coding RNA
lncNAT: Long non-coding natural antisense transcripts
PRC2: Polycomb repressive complex 2
HOTAIR: HOX ANTISENSE INTERGENIC RNA
miRNA: microRNA
IPS1: INDUCED BY PHOSPHATE STARVATION 1
eTM: Endogenous microRNA target mimic
RNA-seq: High-throughput RNA sequencing technology
LRK: Leucine-rich repeat receptor kinase
LAIR: LRK antisense intergenic RNA
CPC: Coding Potential Calculator
CNCI: Coding Non Coding Index
PLEK: Predictor of long non-coding RNAs and messenger RNAs based on an improved k-mer scheme
PFAM: Protein families database
GO: Gene ontology
qRT-PCR: Quantitative real-time PCR
FPKM: Fragments per kilobase of transcript per million fragments mapped
snRNA: Small nuclear RNA
snoRNA: Small nucleolar RNA
PSTVd: Potato spindle tuber viroid
SRA: Sequence Read Archive
NCBI: National Center for Biotechnology Information
