# Supplementary figures and images for: Genome-wide identification and characterization of long non-coding RNAs related to grain yield in foxtail millet [Setaria italica (L.) P. Beauv.]
Source: BMC Genomics. 2020 Dec 1;21:853. doi: 10.1186/s12864-020-07272-9 (PMC7709324; doi:10.1186/s12864-020-07272-9)

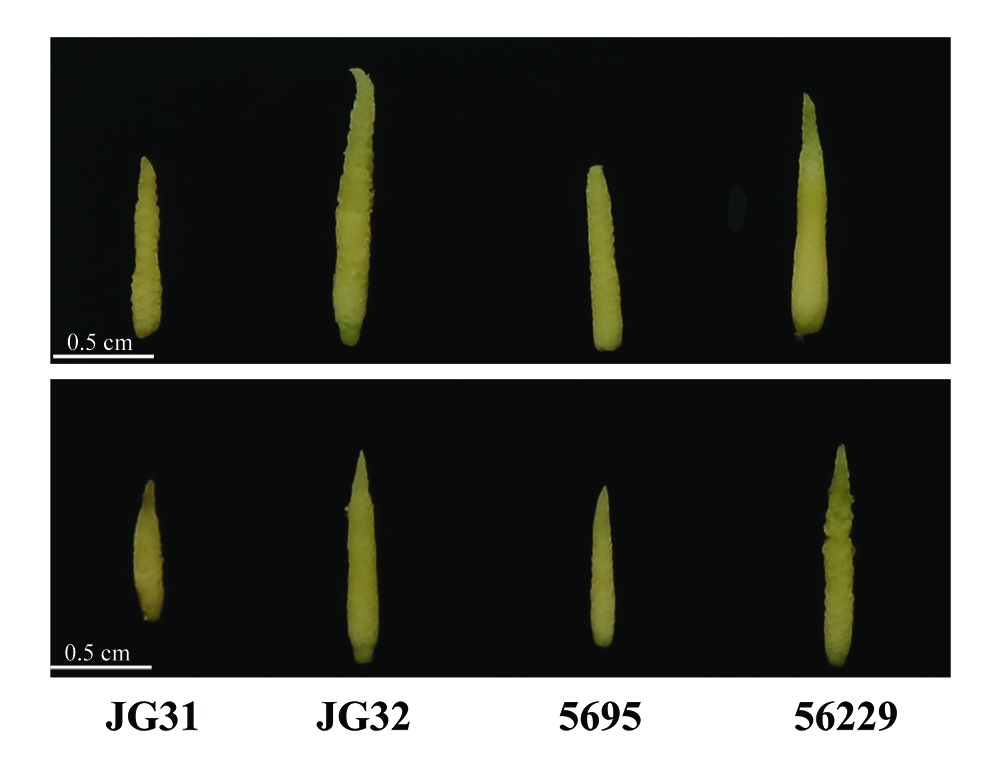

Supplement: Supplementary file 1 — Additional file 1: Fig. S1. The young spikelets of four foxtail millet varieties at booting stage were laid out. [file 12864_2020_7272_MOESM1_ESM.tif]

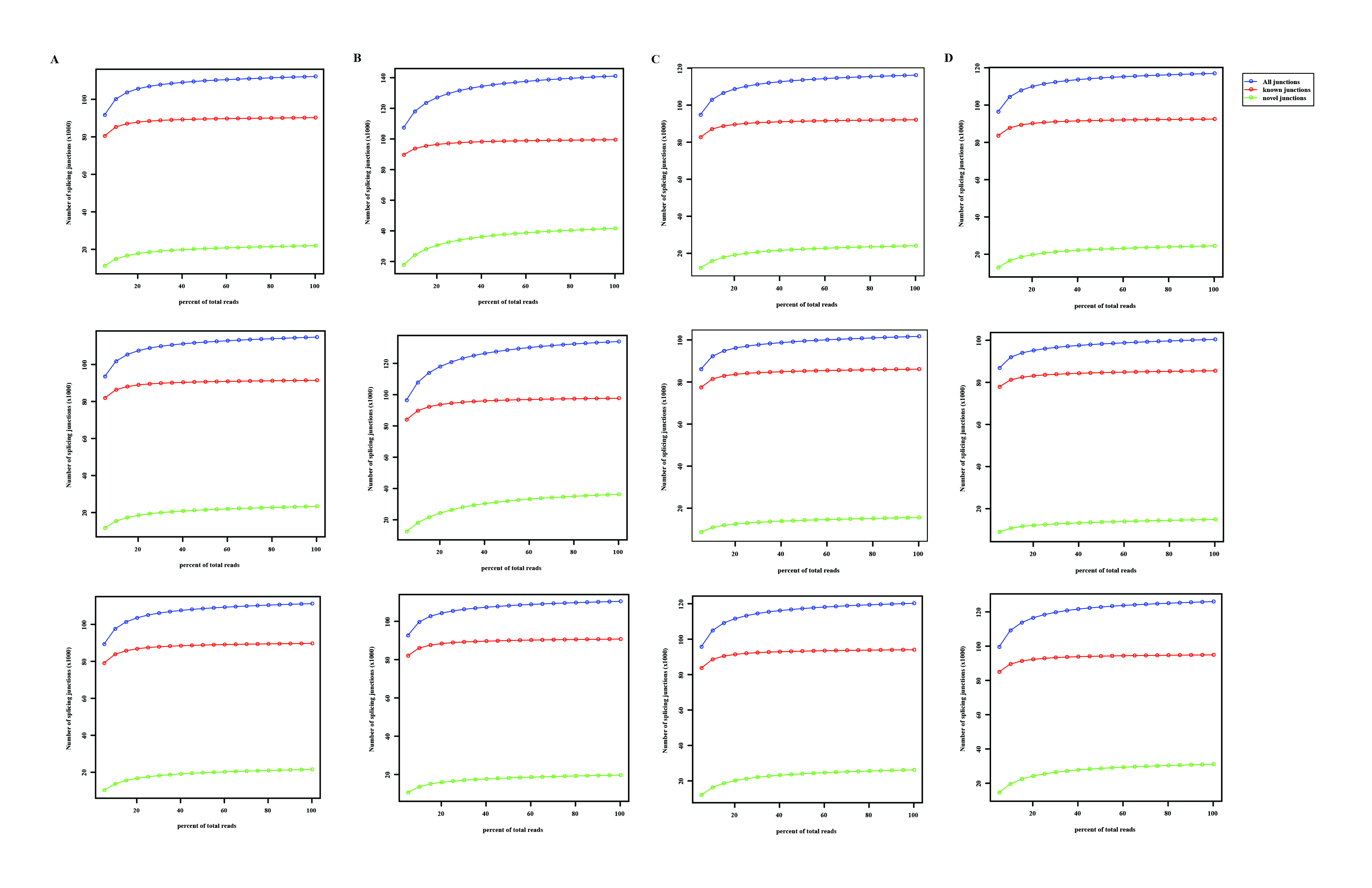

Supplement: Supplementary file 3 — Additional file 3: Fig. S2. The saturation analysis of lncRNAs from four foxtail millet varieties. The three biological replicates of RNA-seq saturation for lncRNAs from JG31 (A), JG32 (B), 5695 (C) and 56229 (D) were shown. X axis is the percentage of total reads resampled. Y axis is the number of detected splicing junctions. [file 12864_2020_7272_MOESM3_ESM.tif]

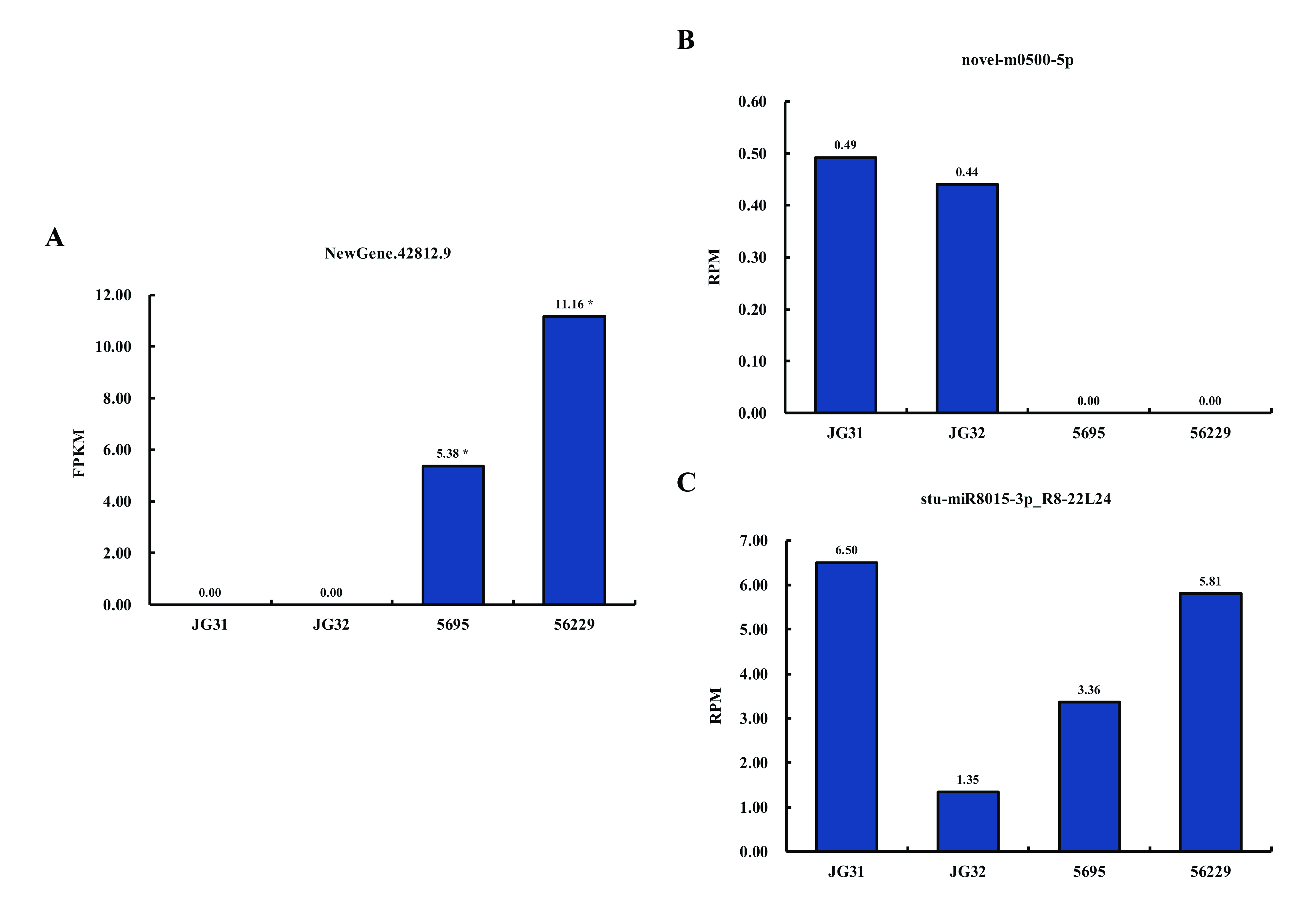

Supplement: Supplementary file 8 — Additional file 8: Fig. S3. The expression levels of two miRNAs and their predicted lncRNA precursor. The lncRNA NewGene.42812.9 (A) was predicted precursor of miRNAs novel-m0500-5p (B) and stu-miR8015-3p_R8-22L24 (C). FPKM, fragments per kilobase of transcript per million fragments mapped. RPM, reads per kilobase of transcript per million mapped. [file 12864_2020_7272_MOESM8_ESM.tif]

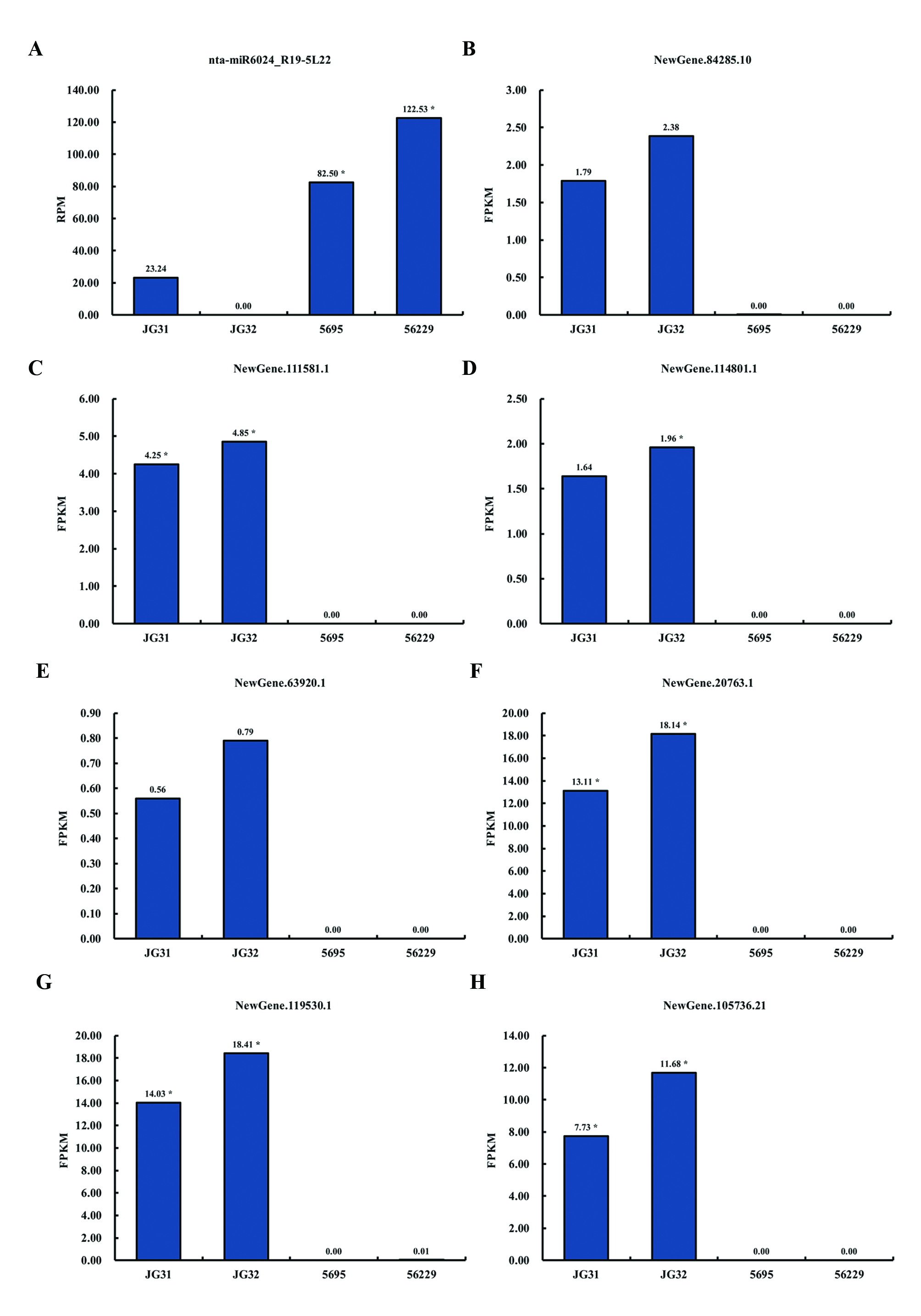

Supplement: Supplementary file 9 — Additional file 9: Fig. S4. The expression levels of miRNA and its predicted target mimics. The lncRNAs NewGene.84285.10 (B), NewGene.111581.1 (C), NewGene.114801.1 (D), NewGene.63920.1 (E), NewGene.20763.1 (F), NewGene.119530.1 (G) and NewGene. 105,736.21 (H) were predicted target mimics of miRNA nta-miR6024_R19-5 L22 (A). FPKM, fragments per kilobase of transcript per million fragments mapped. RPM, reads per kilobase of transcript per million mapped. [file 12864_2020_7272_MOESM9_ESM.tif]
