# Supplementary material for: Genome-wide identification and characterization of long non-coding RNAs related to grain yield in foxtail millet [Setaria italica (L.) P. Beauv.]
Source: BMC Genomics. 2020 Dec 1;21:853. doi: 10.1186/s12864-020-07272-9 (PMC7709324; doi:10.1186/s12864-020-07272-9)
Supplement: Supplementary file 2 — Additional file 2: Table S1. Yield performance of four foxtail millet varieties used in this study. [file 12864_2020_7272_MOESM2_ESM.docx]

**Additional file 2: Table S1.** Yield performance of four foxtail millet varieties used in this study.

| Year | Variety | Yield in Tangshan (kg/0.0013 hectare) | | | Yield in Xingtai (kg/0.0013 hectare) | | | Average  (kg/0.0013 hectare) | Compared to JG31 (±%) | Compared to JG32 (±%) |
| --- | --- | --- | --- | --- | --- | --- | --- | --- | --- | --- |
|  |  | Ⅰ | Ⅱ | Ⅲ | Ⅰ | Ⅱ | Ⅲ |  |  |  |
| 2018 | JG31 | 8.82 | 7.84 | 7.47 | 7.97 | 9.17 | 8.46 | 8.29 |  |  |
|  | JG32 | 8.35 | 8.16 | 8.24 | 8.15 | 8.98 | 8.41 | 8.38 |  |  |
|  | 5695 | 11.32 | 10.69 | 10.61 | 9.19 | 9.27 | 9.83 | 10.15 | 22.48 | 21.17 |
|  | 56229 | 10.28 | 10.60 | 10.33 | 9.32 | 9.17 | 10.21 | 9.99 | 20.47 | 19.18 |
| 2019 | JG31 | 6.22 | 6.42 | 6.10 | 8.76 | 7.63 | 8.41 | 7.26 |  |  |
|  | JG32 | 6.01 | 6.98 | 6.19 | 8.24 | 9.13 | 8.26 | 7.47 |  |  |
|  | 5695 | 7.43 | 8.37 | 7.46 | 10.68 | 11.25 | 9.80 | 9.17 | 26.30 | 22.72 |
|  | 56229 | 6.05 | 8.67 | 7.37 | 10.78 | 9.49 | 10.08 | 8.74 | 20.44 | 17.03 |
